# Circulating tumour cells in regionally metastatic cutaneous squamous cell carcinoma: A pilot study

**DOI:** 10.18632/oncotarget.9946

**Published:** 2016-06-11

**Authors:** Tia Morosin, Bruce Ashford, Marie Ranson, Ruta Gupta, Jonathan Clark, N. Gopalakrishna Iyer, Kevin Spring

**Affiliations:** ^1^ Graduate School of Medicine, University of Wollongong, Wollongong, NSW 2522, Australia; ^2^ School of Biological Sciences, University of Wollongong, Wollongong, NSW 2522, Australia; ^3^ Illawarra Health and Medical Research Institute (IHMRI), Wollongong, NSW 2522, Australia; ^4^ Illawarra and Shoalhaven Local Health District (ISLHD), Wollongong, NSW 2522, Australia; ^5^ Centre for Oncology Education and Research Translation (CONCERT), Liverpool, NSW 2170, Australia; ^6^ Department of Tissue Pathology and Diagnostic Oncology, Royal Prince Alfred Hospital, Camperdown, NSW 2050, Australia; ^7^ The University of Sydney, Sydney, NSW 2006, Australia; ^8^ Sydney Head and Neck Cancer Institute, Department of Head and Neck Surgery, The Chris O'Brien Lifehouse, Camperdown, NSW 2050, Australia; ^9^ Central Clinical School, The University of Sydney, Sydney, NSW 2006, Australia; ^10^ South West Clinical School, Faculty of Medicine, The University of New South Wales, Sydney, NSW 2052, Australia; ^11^ Singhealth/Duke-NUS Head and Neck Center, National Cancer Center Singapore (NCCS), 169610, Singapore; ^12^ Medical Oncology Group, Ingham Institute for Applied Medical Research, Liverpool, NSW 2170, Australia; ^13^ Liverpool Clinical School, Western Sydney University, Liverpool, NSW 1871, Australia

**Keywords:** circulating tumour cells (CTCs), metastases, cutaneous, squamous cell carcinoma (SCC), head and neck

## Abstract

**Background:**

Circulating tumour cells (CTCs) are increasingly being used in the surveillance of cancer, allowing for potential early detection and real-time monitoring of disease progression. The presence of CTCs in patients with metastatic cutaneous head and neck squamous cell carcinoma (cHNSCC) has not been evaluated.

**Results:**

CTCs were detected in eight of ten patients with regional metastatic cHNSCC (80%; range 1–44 cells/9 mL blood). CTMs were detected in three of ten patients (30%, range 1–4 cells/9 mL blood).

**Methods:**

Preoperative blood samples from ten patients with nodal metastases from cutaneous squamous cell carcinomas (cSCC) were analyzed using the IsoFlux^TM^ System for the detection and enumeration of CTCs and circulating tumour microemboli (CTMs).

**Conclusions:**

For the first time CTCs have been detected in patients with nodal metastases from cHNSCC. Further work is required to understand their prognostic significance and potential to directly influence clinical practice.

## INTRODUCTION

Australia has the highest incidence of skin cancer worldwide, with two in every three Australians developing this debilitating disease in their lifetime. Australians are four times more likely to develop skin cancer than any other form of cancer [1, 2]. Non-melanoma skin cancer (NMSC) accounts for over 430,000 new cases each year; with 138,000 (20–25%) being squamous cell carcinoma (cSCC) [2, 3]. In 2010 alone, there were 445 deaths as a result of NMSC, predominately SCC [4].

UV radiation is the most significant risk factor for cSCC, with the majority of NMSCs located on sun-exposed areas of the body, particularly the head and neck (70%) [1, 5]. cSCC accounts for 20% of all head and neck malignancies [6].

Treatment of primary lesions includes surgical resection, with the addition of adjuvant radiation therapy in advanced disease [7]. Currently, adjuvant chemotherapy is employed infrequently outside of a trial setting. Despite aggressive first line treatment, approximately 5% of cSCC cases develop nodal metastases [7]. Whilst relatively uncommon, 70% of metastases occur within the first 12 months following treatment of the primary lesion, with a poor prognosis and 5-year overall survival rate of 11% in patients with distant metastases [7–10].

The early identification of patients at higher risk for metastatic spread would help rationalise the need for surveillance. Currently, other than traditional histopathologic assessment [11], little is understood about the potential for metastasis in cSCC.

Carcinomas spread by lymphatic and/or vascular infiltration giving rise to circulating tumour cells (CTCs) that are shed from the primary tumour [12]. These CTCs may exist individually or as microemboli, are readily detectable in the peripheral blood and are increasingly being used in surveillance in breast, lung, colorectal (CRC) and prostate cancer [13]. Subpopulations of tumours may have the ability to enter the vascular space by mesenchymal transition (Epithelial Mesenchymal Transition). Once in the vascular space, tumour cells have the opportunity to metastasize to distant sites (Mesenchymal Epithelial Transition). The detection of the CTCs is a snapshot of this process. As noted by Grisanti [14] CTCs have the potential to provide ‘real-time’ monitoring acting as ‘liquid biopsies’. CTC estimation may allow real-time monitoring of disease progression or reoccurrence and earmark some patients for a change or reintroduction of treatment. No studies have as yet focused on CTCs as markers for primary or metastatic cSCC. This pilot study aimed to preoperatively identify the presence of CTCs in patients with nodal metastases from cSCC in the head and neck prior to surgery.

## RESULTS

A summary of patient characteristics and results is provided in Table 1. All patients had previously undergone primary tumour resection and had no evidence of distant metastatic disease (M0). The IsoFlux^TM^ System was able to detect the presence of CTCs in eight of ten patients (80%) with cell counts ranging from 1–44 in 9 mL of blood. In addition, CTC microemboli clusters (CTMs) were detected in three patients (30%), with the highest CTC and CTM counts attributed to the same patient. All ten patients were treated with curative intent by surgery, and eight of ten received adjuvant radiotherapy. Two patients succumbed to their disease within the study period. Both of these patients had undetectable CTCs and CTMs at the time of blood collection.

**Table 1:** Patient summary and circulating tumour cell and microemboli results

								Treatment	
Patient	Age	Sex	Pathology	Nodal stage	Histopathology grade	CTC count	CTM count	Surgery	Radiation therapy
1	85	M	cSCC	N2b	G3	4	2	Yes	Adjuvant
2	67	M	cSCC	N2a	G3	0	0	Yes	Adjuvant
3	69	M	cSCC	N2a	G3	19	0	Yes	Adjuvant
4	77	M	cSCC	N2a	G3	14	1	Yes	Adjuvant
5	62	M	cSCC	N2a	G3	44	4	Yes	Adjuvant
6	77	M	cSCC	N1	G3	14	0	Yes	Adjuvant
7	88	M	cSCC	N2b	G3	1	0	Yes	Prior
8	78	M	cSCC	N1	G2	4	0	Yes	Prior
9	54	M	cSCC	N2b	G1	1	0	Yes	Adjuvant
10	66	M	cSCC	N3	G3	0	0	Yes	Adjuvant

## DISCUSSION

Detectable CTCs have shown implications for disease-free survival, overall survival, risk of local reoccurrence and risk of metastases in subgroups of other solid tumour groups [15]. However no studies have as yet focused on CTCs as markers for primary or metastatic disease in patients with cSCC. This is the first study to examine the presence of CTCs in metastatic cSCC.

CTCs were identified in eight of ten patients with metastatic cSCC using the IsoFlux^TM^ System. We also identified circulating tumour microemboli clusters (CTM) in three of ten patients.

Numerous prospective studies have now shown a significant association between CTCs and worsening metastatic disease, with baseline CTCs acting as strong predictors of decreased survival, independent of other factors in breast, prostate and colorectal cancer [13]. Notably, Jatana [15] demonstrated improved disease-free survival in patients with no detectable CTCs in mucosal HNSCC. However, in another study, the absence of CTCs preoperatively did not necessarily portend *a favourable* outcome in mucosal HNSCC [16]. These authors also reported that the only patient (of 16) where no CTCs were identified later succumbed to locoregional and distant metastatic disease. Interestingly, in our series, there has been no correlation between the presence of CTCs and patient outcome in the early stages of follow-up. Indeed, during the 12-month follow-up period, both patients who died (5 months and 8 months post surgery) had no recordable CTCs in their preoperative blood sample. Both patients had recurrent post radiotherapy disease at the time of CTC sampling.

Whilst we were successful in detecting CTCs, the levels detected were quite small in comparison to other studies [15]. As noted by Grisanti [14], this may reflect a genuine low count due to the extremely rare nature of CTCs in the blood (estimations of 1 in 100 million to 1 in one billion blood cells). Alternatively, it may represent a falsely low count as a result of the absence of Ep-Cam and cytokeratin expression on CTCs displaying greater mesenchymal morphology [14].

Notably, an additional patient enrolled in the study was excluded from analysis due to deviation from the experimental protocol because blood was collected intra-operatively. This collection produced interesting results with this patient having much higher CTCs/CTMs counts detected (86 and 25, respectively). These findings echo those of previous animal models which have shown correlation between disseminated cancer cells as a result of surgical manipulation of tumour, and is paralleled by human studies which have displayed increased counts of CTCs intraoperatively compared to pre- and post-operative counts in surgical removal of colorectal liver metastases [17, 18]. Once again, this highlights the effect of surgery on CTC number, although the significance of this is elusive.

We have identified the presence of Ep-CAM and cytokeratin expressing cells in the circulation of patients with metastatic cSCC. A broadening of CTC markers in a positive selection model using mesenchymal and novel biomarkers may show higher levels, and may add to our understanding of the metastatic process in cSCC. As cSCC is the most common malignancy with metastatic potential, the ability to identify the small percentage of patients where metastases occur would have enormous public health implications, particularly in regions with high solar exposure. The establishment of CTCs as markers for metastatic disease also has the potential to directly inform clinical practice by early identification of recurrence and the development of individualized treatment pathways for patients with high-risk disease.

## MATERIALS AND METHODS

### Patient selection

Ethics approval was obtained from the UOW/ISLHD Human Research Ethics Committee (HE14/397). Inclusion criteria were patients who were candidates for surgical treatment of metastatic cSCC affecting the lymph nodes of the parotid and/or neck. Eleven patients were enrolled, with one patient excluded from analysis due to a major deviation from the experimental protocol.

### Blood collection

Peripheral blood samples of 9mL were collected and drawn into anti-coagulant K_3_ EDTA coated tubes (Vacuette 455036; Greiner Bio-One, Interpath Services, VIC, Australia) at the time of cannulation immediately pre-operatively. Blood samples were kept at room temperature and processed within 24 hours of collection, using the IsoFlux^TM^ System according to manufactures instructions and protocol established by Harb [19].

### Sample preparation and CTC isolation

White cell fraction (WCC) was isolated using LeucoSep tubes (Greiner Bio-One, Interpath Services, VIC, Australia) prepared with 15.2 mL of Ficoll-Paque PLUS (GE Healthcare, NSW, Australia), recovered, then res-suspended in phosphate-buffered saline (PBS) binding buffer. Pre-conjugated immunomagnetic beads (with Anti-EpCAM antibodies; CTC Enrichment kit Fluxion Biosciences Inc.) were added and the sample incubated on a rotator at 4°C for 1.5 hours. Samples were loaded into the microfluidic cartridge of the IsoFlux^TM^ System and processed for 45 minutes, with magnetically labelled target cells captured, isolated and recovered for further processing.

### CTC enumeration and imaging

Recovered cells were fixed (PBS containing 1.8% formaldehyde), washed and blocked with 10% normal donkey serum, then stained with anti-CD45 antibody (CTC Enumeration Kit, Fluxion Biosciences, Inc.), Cy3-conjugated secondary antibody, and permeabilised with 0.1% Triton X-100. Following this, cells were further stained with FITC conjugated anti-cytokeratin antibody (CK; CTC Enumeration Kit, Fluxion Biosciences Inc.), mounted with Hoechst 33342 (Mounting Media; CTC Enrichment Kit, Fluxion Biosciences Inc.), transferred to a SensoPlate Glass-Bottom Multiwell Plate (Grenier Bio-One, Interpath Services, VIC, Australia) and stored in 4° for up to 2 weeks for imaging. As per protocol, cells were counted as CTCs if they were CK+, and CD45–, nucleated, and morphologically intact. Imaging was performed using an inverted Olympus IX71 microscope (Olympus Australia Pty. Ltd., Victoria, Australia) and Proscan II automatic scanning (Prior Scientific Inc., Massachusetts, USA).
